# Low-intensity pulsed ultrasound prevents muscle atrophy induced by type 1 diabetes in rats

**DOI:** 10.1186/s13395-017-0145-7

**Published:** 2017-12-22

**Authors:** Liang Tang, Nan Li, Wenqi Jian, Yiting Kang, Bo Yin, Shuxin Sun, Jianzhong Guo, Lijun Sun, Dean Ta

**Affiliations:** 1 Institute of Sports Biology, Shaanxi Normal University, Xi’an, 710119 China; 2 Leisure Management College, Xi’an Eurasia University, Xi’an, China; 3 Department of Electronic Engineering, Fudan University, Shanghai, 200433 China; 4 Shaanxi Key Laboratory of Ultrasonics, Shaanxi Normal University, Xi’an, China; 5 Key Laboratory of Medical Imaging Computing and Computer Assisted Intervention (MICCAI) of Shanghai, Shanghai, China

**Keywords:** Type 1 diabetes, Low-intensity pulsed ultrasound (LIPUS), Muscle atrophy, MSTN/Akt/mTOR&FoxO1 signaling pathways

## Abstract

**Background:**

Type 1 diabetes mellitus (T1DM) induces serious skeletal muscle atrophy. Low-intensity pulsed ultrasound (LIPUS) is a common treatment for skeletal muscle injury and is effective in accelerating the rate of muscle growth. However, to the best of our knowledge, whether LIPUS can improve skeletal muscle atrophy in type 1 diabetic rats has not been investigated.

**Methods:**

The rats were randomly divided into four groups: the normal control group (NC); the sham-treated diabetic control group (DC); the diabetic, insulin-treated group (DI) as a positive control; and the diabetic LIPUS therapy group (DL). The DL rats were treated with LIPUS (1 MHz, 30 mW/cm^2^) on the gastrocnemius for 20 min/day.

**Results:**

After 6 weeks, the rats in the DC group showed severe muscle atrophy. However, LIPUS significantly improved type 1 diabetes-induced muscle atrophy, as evidenced by significantly enhanced muscle cross-sectional area, muscle mass, and strength. Moreover, compared with the DC group, LIPUS significantly activated Akt and upregulated the expression of mTOR, and LIPUS downregulated the expression of MSTN, its receptor ActRIIB, and FoxO1.

**Conclusions:**

These results indicate that LIPUS improved muscle atrophy induced by type 1 diabetes, and the MSTN/Akt/mTOR&FoxO1 signaling pathway may play a role in this improvement.

## Background

Type 1 diabetes mellitus (T1DM) is characterized by autoimmunity against pancreatic B cells, resulting in their destruction and the patients’ subsequent dependency on lifelong insulin replacement [1–3]. Although the etiology of T1DM has not been fully elucidated to date, genetic, immunological, and environmental factors are now considered key factors of T1DM, an autoimmune disease [4]. T1DM patients have many complications, including cardiovascular, renal, and retinal disorders [5–7]. Among them, skeletal muscle is a major target tissue of diabetic damage [8]. Skeletal muscle is one of the largest organs in the human body and is, quantitatively, the most important tissue involved in maintaining glucose homeostasis under insulin-stimulated conditions [9]. Type 1 diabetic subjects without insulin treatment display a dramatic loss of muscle [10], which leads to a higher blood glucose concentration, resulting in a vicious cycle.

Low-intensity pulsed ultrasound (LIPUS) is a common treatment for skeletal muscle injury [11]. LIPUS is a type of mechanical energy that is transmitted through and into living tissues in the form of acoustic pressure waves [12]. In muscle tissues, LIPUS can stimulate the proliferation of myogenic precursor cells and myogenic cells [11, 13]. In muscle treated with ultrasound, an aligned and more regular disposition of collagen fibers and myotubes is observed, enabling increased functionality [11, 14]. LIPUS, as an inexpensive, safe, and noninvasive treatment, has been widely demonstrated to accelerate the rate of muscle growth [13]. Moreover, our latest results show that LIPUS promotes exercise-induced muscle hypertrophy [15]. However, whether LIPUS can improve skeletal muscle atrophy in type 1 diabetic rats has not been previously investigated.

Myostatin (MSTN) is a member of the transforming growth factor-β (TGF-β) superfamily of secreted growth factors and is a negative regulator of skeletal muscle development [16]. Loss of MSTN function leads to a dramatic and specific increase in skeletal muscle mass [17]. Nevertheless, mice with muscle atrophy show increased MSTN expression [18]. MSTN has been implicated in the regulation of skeletal muscle mass and has emerged as a novel therapeutic target for treating several degenerative muscle diseases and muscular dystrophy [19, 20]. MSTN blockade with a fully human monoclonal antibody induces skeletal muscle hypertrophy and prevents atrophy induced by immobilization, hindlimb suspension, and dexamethasone [21].

Protein kinase B (Akt), a serine/threonine kinase, is recruited to the membrane by an interaction with phosphoinositide docking sites for full activation [22]. Activated Akt mediates downstream responses, including cell survival, growth, proliferation, cell migration, and angiogenesis, by phosphorylating a range of intracellular proteins [23]. Forkhead box protein O1 (FoxO1), a member of the FoxO forkhead-type transcription factors, can suppress an increase in skeletal muscle mass [24, 25]. The activation of Akt has been shown to phosphorylate FoxO1, and then, the phosphorylated FoxO1 proteins, sequestered in the cytosol, are unable to transcribe genes involved in the atrophy process [26, 27]. The Akt/mammalian target of rapamycin (mTOR) pathway is implicated in numerous cellular processes ranging from cell growth and survival to the promotion of angiogenesis [28]. Briefly, the Akt/mTOR&FoxO1 signal pathways play a key role in increasing protein synthesis and decreasing protein degradation in muscles [29].

In this study, the effect of LIPUS therapy on skeletal muscle atrophy in type 1 diabetic rats was investigated, and the underlying mechanisms were later explored by analyzing the gene and protein expression of MSTN, its receptor, activin receptor type IIB (ActRIIB), Akt, p-Akt, FoxO1, and mTOR.

## Methods

### Animals

Fifty male Sprague Dawley rats (200–240 g) were obtained from the Laboratory Animal Breeding and Research Center of Xi’an Jiaotong University (Xi’an, China) and were housed in a controlled room (22 ± 2 °C, 60 ± 5% humidity, and 12-h light/dark cycle). All experiments were conducted with the approval of the Animal Ethical Committee of Shaanxi Normal University and in accordance with the Guide for the Care and Use of Laboratory Animals published by the US National Institutes of Health (NIH publication no. 85-23, revised 1996).

### Induction of T1DM model

After 5 days of acclimation, the rats were randomly assigned to either the normal control group (NC, *n* = 8) or the T1DM model group (T1DM, *n* = 42). T1DM was induced by a single intraperitoneal injection of streptozotocin (STZ, Sigma, St. Louis, MO) at 60 mg/kg body weight, prepared in citrate buffer (0.1 M, pH = 4.5). An equivalent dose of sterile citrate buffer solution was injected into the NC rats. After STZ administration, the induction of the T1DM model was confirmed by measuring the blood glucose levels in tail vein blood samples on the first, third, seventh, and tenth days. The rats with a blood glucose concentration greater than or equal to 16.7 mmol/L (300 mg/dL) were considered to be the qualified T1DM models. Eleven rats subsequently received daily 20 mg/kg STZ injections for 3 days. During the modeling experiment, all rats were transferred to metabolic cages in which the amount of food intake and water consumed were precisely recorded between 9:00 AM and 21:00 PM. Once T1DM was induced, the diabetic rats were randomly allocated into three groups: sham-treated diabetic control rats (DC + Sham, DC, *n* = 8), T1DM rats treated with an insulin injection (DC + INS, DI, *n* = 8), and T1DM rats treated with LIPUS (DC + LIPUS, DL, *n* = 8). Each group was randomly allocated two rats received more than one STZ injections.

### Insulin dose

The rats in the DI group were given a subcutaneous insulin injection, 6–8 U/day at 8:00–10:00 AM and 9:00 PM. The dosage of insulin was adjusted according to the non-fasting glucose level before each insulin injection.

### LIPUS therapy

**Fig. 1:**
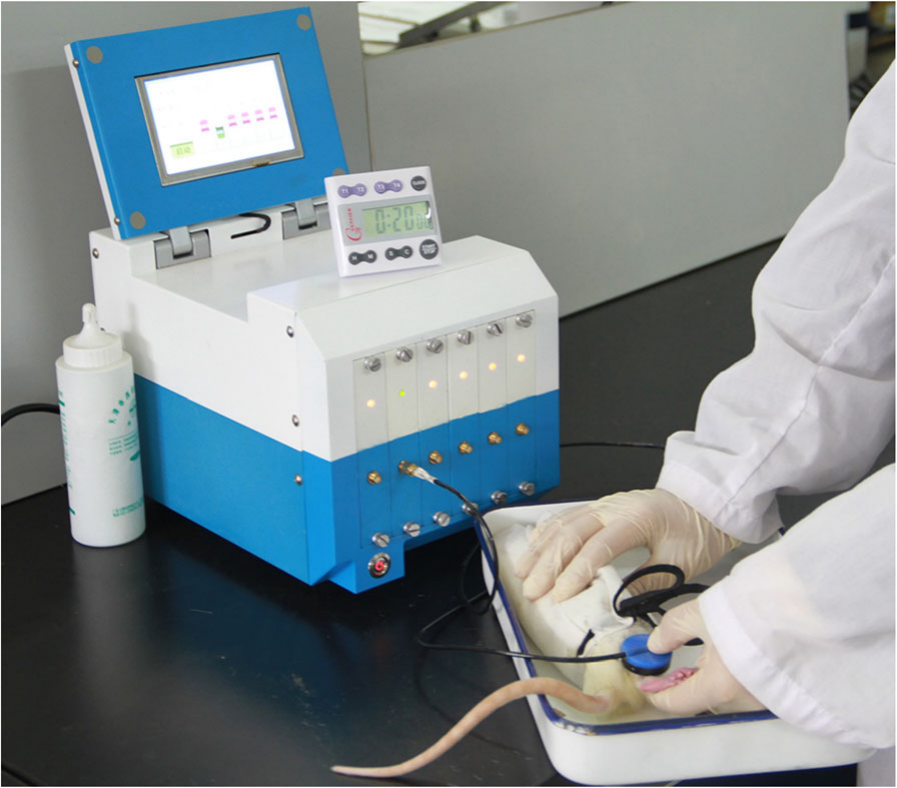
LIPUS instrument. The LIPUS device produced a 2000-μs burst of 1-MHz acoustic sine waves repeating at 100 Hz with a spatial-averaged temporal-averaged (SATA) intensity of 30 mW/cm^2^. In the LIPUS group, the bilateral calf muscles of the rats were exposed to LIPUS for 20 min/day for 6 weeks

A LIPUS device, provided by Shanghai Acoustics Laboratory, Chinese Academy of Sciences, produced a 2000-μs burst of 1-MHz acoustic sine waves repeating at 100 Hz with a spatial-averaged temporal-averaged (SATA) intensity of 30 mW/cm^2^. The DL group rats were treated with LIPUS at the bilateral calf muscles after shaving, for 20 min/day, 6 days/week, for 6 weeks (Fig. 1). The rats were placed in a homemade breathable cloth bag with food. Because rats prefer to dig holes, they can get into the cloth bag easily. The whole superficial calf muscle received LIPUS treatment. Since the rats’ heads were gently touched, and the procedure accompanied their feeding time, the rats were in a relaxed state during the entire therapy. The DC group was treated similarly as the DL group, except that the power of the LIPUS device was not turned on.

### Body weight, blood glucose, and sample preparation

Body weights and fasting blood glucose concentrations were obtained once a week and once every 2 weeks, respectively. The blood was collected from the tail vein. Rats were sacrificed with an overdose of diethyl ether after 6 weeks of treatment. The quadriceps, gastrocnemius, and soleus were dissected and weighed.

### Oral glucose tolerance test (OGTT)

After an overnight fast, rats were given an oral dose of glucose (2 g/kg of body weight). Blood samples were obtained from the tail vein at 0, 30, 60, 90, and 120 min after the glucose challenge. Blood glucose concentrations were measured by an eBsensor EB G glucose meter (Visgeneer, Inc., Taiwan, China).

### Grip tests

Grip tests were performed for the two forelimbs with a grip strength meter (YLS13A, Anhui Zhenghua Bioinstrumentation Co., Ltd., Huaibei, Anhui, China). The rats were tested three times in succession without rest. The values of the three peak grip strengths were averaged for each rat.

### Morphometric analysis

The gastrocnemius muscles were dissected rapidly. Muscle tissues were weighed and fixed with 4% polyaldehyde for 24 h. Serial 8–10 μm transverse sections, made with a cryostat, were mounted on silanized slides (Dako, Tokyo, Japan). After fixation, the muscle tissue was embedded in paraffin, sectioned, and stained with hematoxylin and eosin. A histophysiological evaluation was performed under a light microscope. The average areas of the myofibers in each of the images were measured at ×200 magnification using the image analysis software Image-Pro Plus 6.0.

### Western blot analysis

The protein concentrations in the gastrocnemius muscles were measured using the BCA protein assay kit. Equal amounts of total protein were electrophoresed in SDS polyacrylamide gels (8–12%) and transferred to a nitrocellulose membrane. The immunoblots were incubated with primary antibodies (Cell Signaling Technology, Inc., Beverly, MA, USA) overnight at 4 °C followed by incubation with the corresponding secondary antibodies (Cell Signaling Technology, Inc., Beverly, MA, USA) at room temperature for 1 h. Immunoreactive proteins were detected with the enhanced chemiluminescence system (ECL; Amersham). Protein bands were captured using the Azure Biosystems C300 imaging system (Azure Biosystems, Inc., USA), and optical densities were quantified using Bio-Rad Quantity One software. GAPDH was used as the internal loading control. The following secondary antibodies were used: MSTN (EPR4567(2), ab124721) and ActRIIB (EPR10739, ab180185) from Abcam and GLUT4 (2213S), Akt (9272S), mTOR (2972S), FoxO1 (2880S), and phosphor Akt (ser473, 9271S) from Cell Signaling Technology.

### Quantitative real-time PCR analysis

Total RNA from gastrocnemius muscles (100 mg) was prepared using TRIzol Reagent (Invitrogen Corporation, California, USA) according to the manufacturer’s protocol. The total RNA was treated with DNase I (Invitrogen Corporation California, USA) to remove contaminating genomic DNA and was reverse transcribed using the Prime Script™ II first-Strand cDNA Synthesis Kit (Takara Bio, Otsu, Japan). For quantitative real-time PCR, 0.8 mL of first-strand cDNA was used in a total volume of 10 mL, containing 5 mL of SYBR Green PCR Master Mix (TaKaRa Biotechnology Co., Ltd., Dalian, China), 0.4 mL of each primer, and 3.4 mL of ddH_2_O. The primer sequences and expected product size for the genes amplified are listed in Table 1 (all primers were synthesized by Sangon Biotech Co., Ltd. Shanghai, China). The amplification program consisted of 1 cycle for 1 min at 95 °C followed by 40 cycles of 94 °C for 15 s, 60 °C for 15 s, and 72 °C for 30 s. These reactions were performed with a real-time CFX96 amplifier (Bio-Rad Laboratories, Inc., USA). Next, the RNA levels were calculated based on the 2^ΔΔCT^ method. GAPDH was used as a housekeeping gene.

**Table 1:** Primer design for the RT-PCR assay

Gene	Primer	PCR product length (bp)	Sequence 5′ to 3′	Tm (°C)
MSTN	F	423	GATTATCACGCTACCACG	58.9
	R		ATTCAGCCCATCTTCTCC	
ActRIIB	F	163	GCAGTCGTGGCAGAGTGAGCG	51.7
	R		CTTGAGGTAATCCGTGAGGGAGC	
Akt	F	269	TAGGCATCCCTTCCTTACAG	55.8
	R		GCCCGAAGTCCGTTATCT	
FoxO1	F	459	TCAAGGATAAGGGCGACAG	54.4
	R		GTGGATACACCAGGGAATG	
mTOR	F	368	CTGGCTTCCAACCCTAAA	53.5
	R		TCTCCAAATCCCACTCCC	
GLUT4	F	545	GTC ATC AAC GCC CCA CAG	56.8
	R		TCA GGA CAG AAG GGC AAC AG	
GAPDH	F	292	CGACTGTTAGAACTCCCTCA	57.4
	R		CATTGGGGGTAGGAACAC	

*F* forward, *R* reverse

### Statistical analysis

The results are expressed as the mean ± SD. Statistical analyses were performed using SPSS version 20.0 (SPSS Institute, Chicago, IL, USA). One-way analysis of variance was employed for evaluating the existence of differences among the three groups, and once a significant difference was detected, Tukey’s multiple comparisons test was used to determine the significance between every two groups. A *P* value < 0.05 was considered to be statistically significant.

## Results

### Body weight, fasting blood glucose, and OGTT

**Fig. 2:**
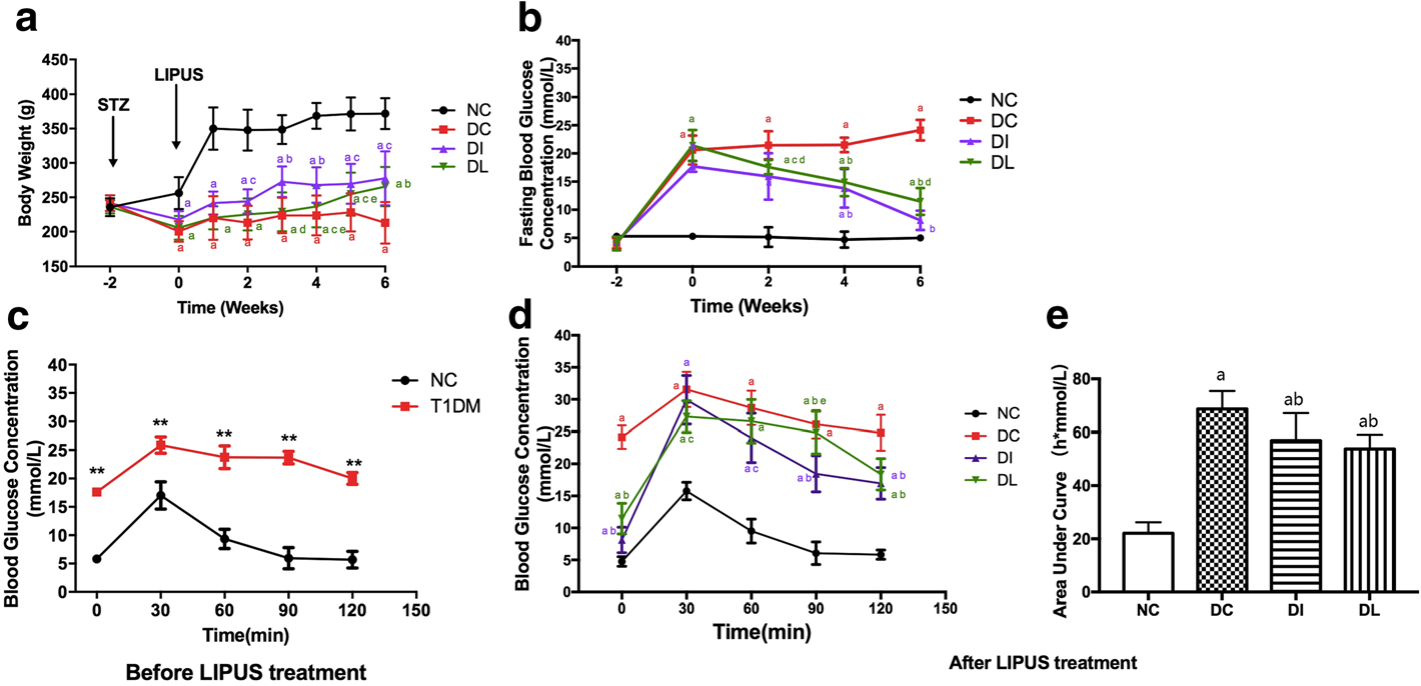
Body weight, fasting blood glucose, and OGTT. **a** Body weights. **b** Fasting blood glucose. **c** OGTT before LIPUS treatment. **d** OGTT after LIPUS treatment. **e** Area under the curve (AUC) after LIPUS treatment. a *P* < 0.01 versus NC group. b *P* < 0.01 versus DC group. c *P* < 0.05 versus DC group. d *P* < 0.01 versus DI group. e *P* < 0.05 versus DI group

The weekly body weights during the whole experimental period are shown in Fig. 2a. After 2 weeks of STZ injection, the body weights of the three diabetic groups were significantly reduced compared with the NC group (*P* < 0.01). The body weights in the NC group continued to be remarkably higher than those in the three diabetic groups during the whole experimental period (*P* < 0.01). LIPUS partially inhibited STZ-induced weight loss from the fourth week (*P* < 0.05), and the body weights of the DI group were significantly increased compared with the DC group from the second week (*P* < 0.05). At the sixth week, there was no significant difference between the DI and DL groups.

The blood glucose levels of the rats in each group during the whole experimental period are depicted in Fig. 2b. After 2 weeks of STZ administration, the blood glucose levels in the three diabetic groups were notably increased to approximately fourfold the NC level. The blood glucose levels in the DC group remained significantly higher than those in the NC group throughout the experiment (*P* < 0.01). LIPUS partially controlled the STZ-induced hyperglycemia from the second week compared with the DC group (*P* < 0.05 at the second week and *P* < 0.01 from the fourth week to the sixth week), and the blood glucose levels in the DL group were still higher than those in the DI group (*P* < 0.01) at the last week.

Oral glucose tolerance tests before and after the LIPUS therapy are presented in Fig. 2c, d. Before the treatment, the blood glucose concentrations in the T1DM group were significantly higher than those in the NC group for the duration of the experiment (*P* < 0.01). However, after 6 weeks of LIPUS treatment, the DC group had significantly higher blood glucose concentrations than the NC group, and this difference persisted for the duration of the experiment (*P* < 0.01). The blood glucose levels in both the DL and DI group descended quickly compared with the DC group. The area under curve (AUC) after the treatment was also calculated. As shown in Fig. 2e, the AUC in the DC group was significantly larger than that in the NC group (*P* < 0.01). However, LIPUS significantly decreased the AUC in T1DM rats (*P* < 0.01), and there was no significant difference between DI and DL group.

### Muscle mass and strength

**Fig. 3:**
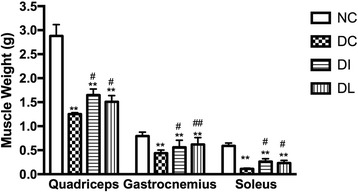
Effect of 6-week exposure to LIPUS on quadriceps, gastrocnemius, and soleus muscle mass. Data are expressed as the mean ± SD (*n* = 8 per group). **P < 0.01 versus NC; ^#^ *P* < 0.05 versus DC group; ^##^ *P* < 0.01 versus DC group

**Fig. 4:**
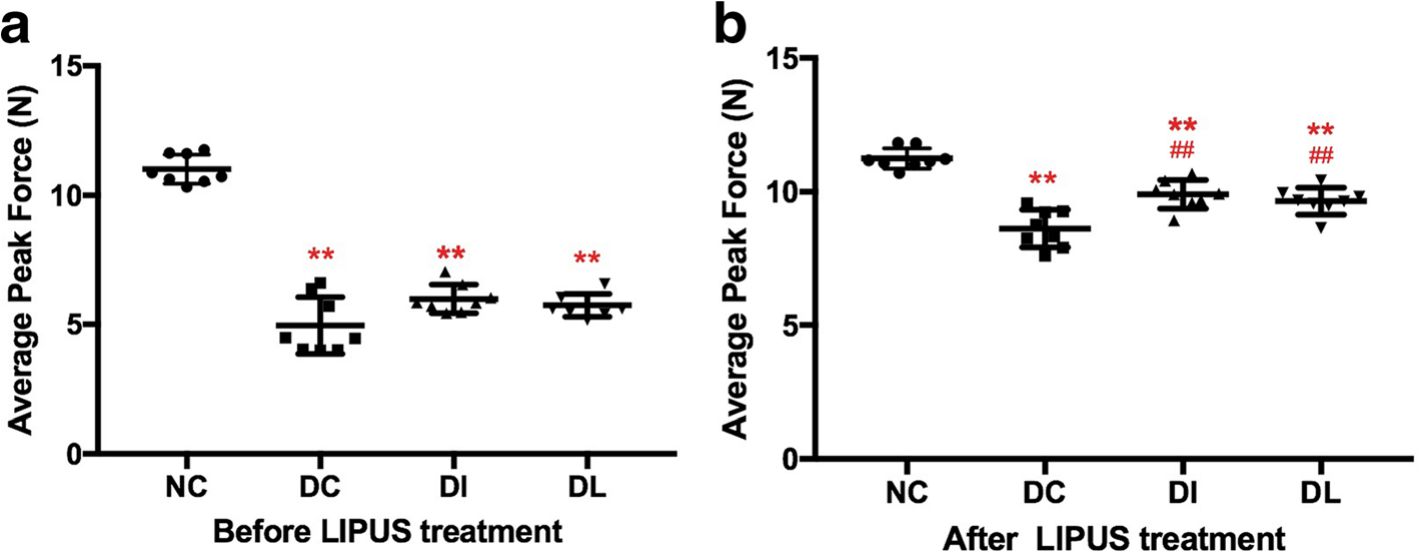
Effect of 6-week exposure to LIPUS on muscle strength. Rats were tested three times in succession without rest. **a** Before LIPUS treatment. **b** After LIPUS treatment. **P < 0.01 versus NC; ^##^ *P* < 0.01 versus DC group

To investigate whether LIPUS could increase skeletal muscle mass and strength, the muscle wet weight and the grip strength of the rats were measured (Figs. 3 and 4). All of the quadriceps, gastrocnemius, and soleus muscle mass of the DC group were lower than that of the NC rats (*P* < 0.01). However, LIPUS significantly increased the quadriceps, gastrocnemius, and soleus muscle mass compared with the DC rats (*P* < 0.05, *P* < 0.01, *P* < 0.05), and there was no significant difference when compared with the DI group.

When the rats were subjected to a grip test before LIPUS treatment, the average peak force of three diabetic groups was lower than that of the NC rats (*P* < 0.01), and there was no significant difference among the three diabetic groups (Fig. 4a). However, after LIPUS treatment, a significant decrease of the average peak grip in the DC rats was still observed compared with the NC rats (*P* < 0.01), while the average peak grip of the DI and DL rats showed an increase greater than that of the DC rats (*P* < 0.01, *P* < 0.01), and there was no significant difference in the grip strength between the DI and DL rats (Fig. 4b). Furthermore, the percent changes from pre-treatment in the DI and DL group (75.66 and 74.21%, respectively) were higher than that in the DC group (69.15%).

### Morphometric analysis

**Fig. 5:**
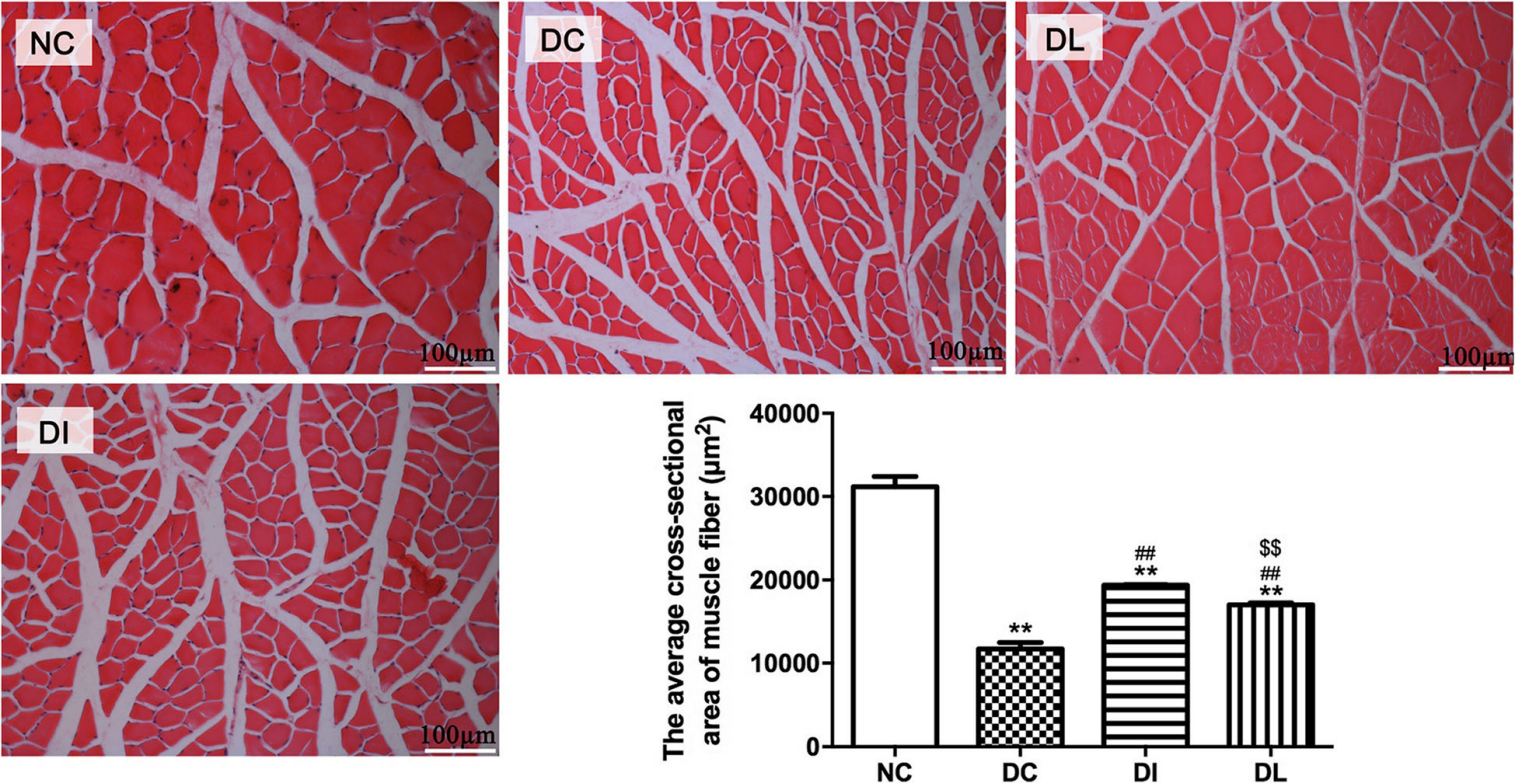
Effect of 6-week exposure to LIPUS on gastrocnemius muscle fiber cross-sectional area. Data are expressed as the mean ± SD (*n* = 8 per group). Each sample represents ten different fields of view; images were taken at ×200 under a light microscope. **P < 0.01 versus NC; ^##^ *P* < 0.01 versus DC group; ^$$^ *P* < 0.01 versus DI group

Morphometric examination of the gastrocnemius muscle was performed (Fig. 5) to test whether the increased muscle mass was accompanied by an increased muscle fiber diameter. The muscle fiber cross-sectional area in the DC group significantly decreased by 62.4% compared with the NC rats (*P* < 0.01). However, the muscle fiber cross-sectional area in the DL group significantly increased by 45.1% compared with the DC rats but was still significantly lower than that of the DI group (*P* < 0.01).

### Gene expression

**Fig. 6:**
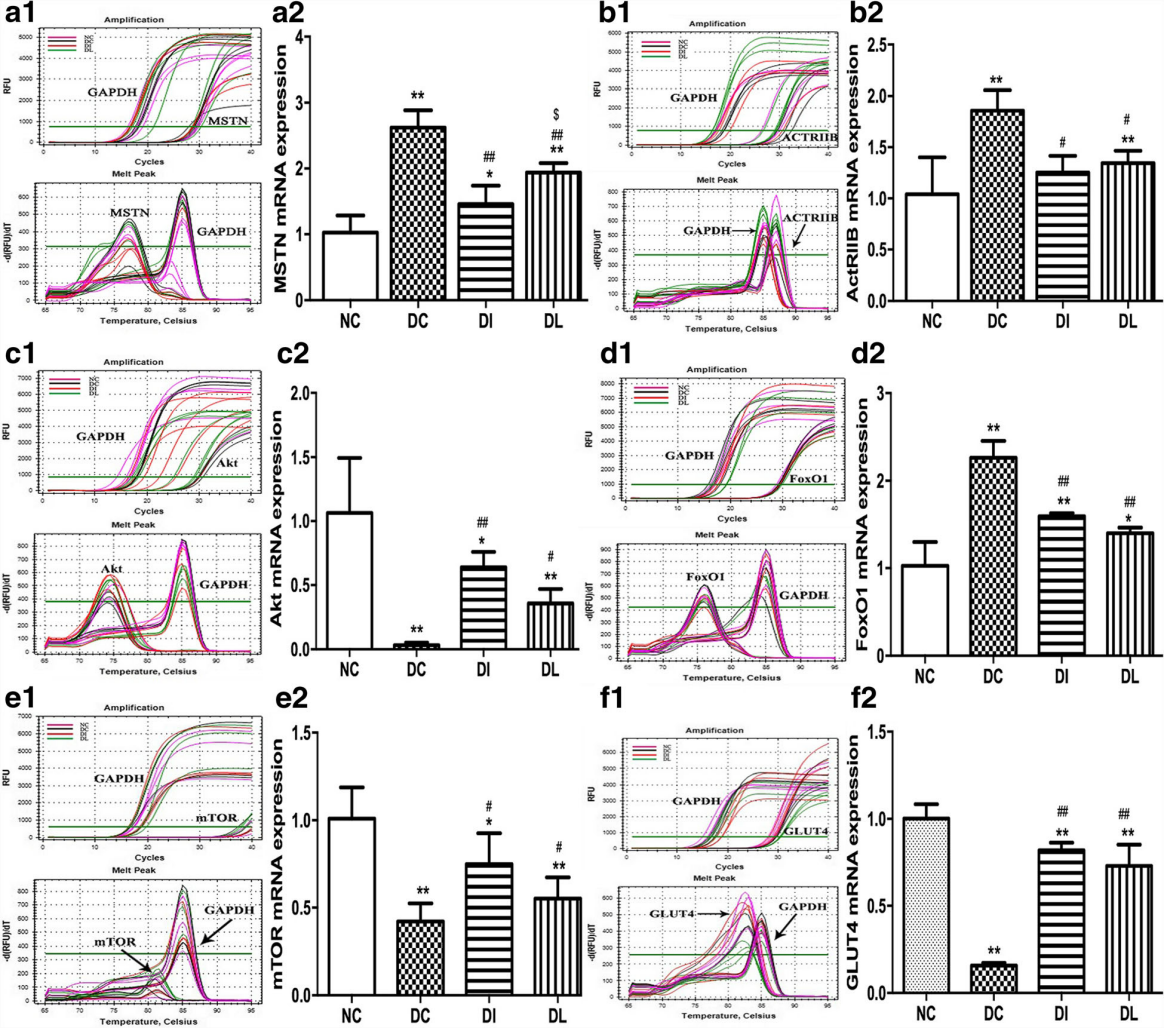
Effect of 6-week exposure to LIPUS on the mRNA expression of MSTN (A1, A2), ActRIIB (B1, B2), Akt (C1, C2), FoxO1 (D1, D2), mTOR (E1, E2), and GLUT4 (F1, F2). A1, B1, C1, D1, E1, and F1 were the amplification curve and dissolution curve. A2, B2, C2, D2, E2, and F2 were the statistic results using the 2^−△△CT^ method. RNA levels were normalized to GAPDH. Data are expressed as the mean ± SD (*n* = 8 per group). *P < 0.05 vs. NC group; **P < 0.01 vs. NC group; ^#^ *P* < 0.05 vs. DC group; ^##^ *P* < 0.01 vs. DC group; ^$^ *P* < 0.05 vs. DI group

The mRNA expression of MSTN, its receptor, ActRIIB, Akt, FoxO1, mTOR, and GLUT4 were analyzed by RT-qPCR (Fig. 6). The mRNA expression of MSTN, ActRIIB, and FoxO1 were significantly upregulated in the DC group compared with the NC group (*P* < 0.01, *P* < 0.01, and *P* < 0.01, respectively), while the mRNA levels of Akt, mTOR, and GLUT4 were significantly decreased in the DC rats compared with the levels of the NC group (*P* < 0.01, *P* < 0.01 and *P* < 0.01, respectively). However, LIPUS significantly decreased the mRNA expression of MSTN, its receptor, ActRIIB, and FoxO1 compared with the DC group (*P* < 0.01, *P* < 0.05, and *P* < 0.01, respectively). LIPUS increased the mRNA expression of Akt, mTOR, and GLUT4 in the DL group compared with the DC rats (*P* < 0.05, *P* < 0.05, and *P* < 0.01, respectively). Moreover, compared with the DI group, the mRNA expression of MSTN in the DL group was significantly higher (*P* < 0.05); however, the expressions of ActRIIB, Akt, FoxO1, mTOR, and GLUT4 in the DL group showed no significant difference compared with the DI group.

### Protein expression

**Fig. 7:**
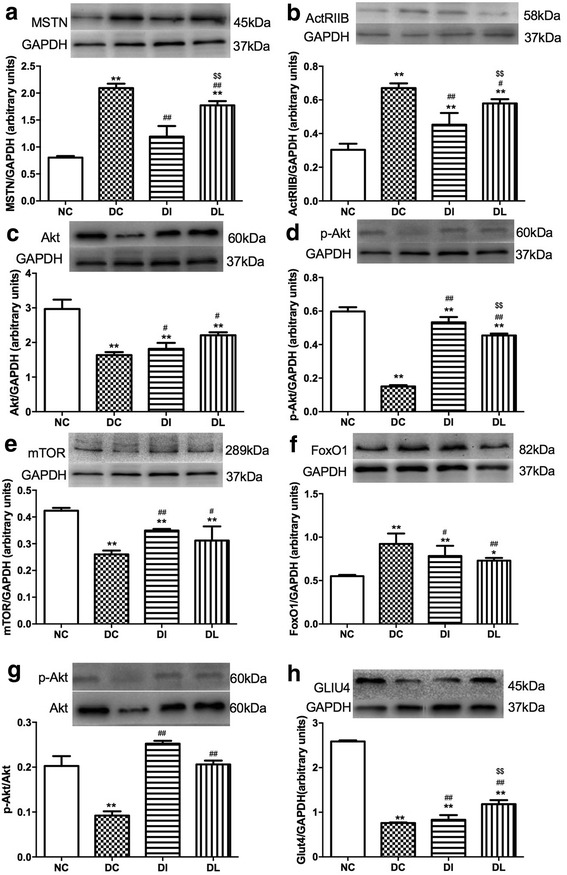
Effects of 6-week exposure to LIPUS on the protein expression of MSTN (**a**), ActRIIB (**b**), Akt (**c**), p-Akt (**d**), mTOR (**e**), FoxO1 (**f**), p-Akt/Akt (**g**), and GLUT4 (**h**). Data are expressed as the mean ± SD (*n* = 8 rats per group). *P < 0.05 vs. NC group; **P < 0.01 vs. NC group; ^#^ *P* < 0.05 vs. DC group; ^##^ *P* < 0.01 vs. DC group; ^$$^ *P* < 0.01 vs. DI group

The protein expression of MSTN, its receptor, ActRIIB, Akt, p-Akt, mTOR, FoxO1, and GLUT4 were examined in the skeletal muscle using western blotting (Fig. 7). MSTN, ActRIIB, and FoxO1 expression levels in the DC group were significantly upregulated compared with the NC group (*P* < 0.01, *P* < 0.01, and *P* < 0.01, respectively), while the protein levels of Akt, p-Akt, mTOR, and GLUT4 were significantly decreased in the DC rats compared with the NC group (*P* < 0.01, *P* < 0.01, *P* < 0.01, and *P* < 0.01, respectively). However, compared with the DC group, 6 weeks of LIPUS treatment significantly decreased the protein expression of MSTN, ActRIIB, and FoxO1 (*P* < 0.01, *P* < 0.05, and *P* < 0.01, respectively) and increased the protein expression of Akt, p-Akt, mTOR, and GLUT4 (*P* < 0.05, *P* < 0.01, *P* < 0.05, and *P* < 0.01, respectively). Compared with the DI group, the expression of MSTN, ActRIB, and GLUT4 in the DL group were higher (*P* < 0.01, *P* < 0.01, and *P* < 0.01, respectively), and the expression of p-Akt was still lower (*P* < 0.01). The protein expression of Akt, mTOR, and FoxO1 in the DL group did not significantly differ from that in the DI group. The p-Akt/Akt ratio of the DC group was lower than that of the NC group (*P* < 0.01), and compared with the DC group, the p-Akt/Akt ratio of the DI and DL groups were higher (*P* < 0.01). There was no significant difference between the DI and DL rats.

## Discussion

Type 1 diabetes mellitus (T1DM) causes skeletal muscle atrophy [2, 10, 30]. LIPUS can increase the differentiation of muscular lineage cells and favor tissue regeneration [13, 31, 32]. Therefore, we investigated whether LIPUS could improve T1DM-induced skeletal muscle atrophy. Our results demonstrated that LIPUS therapy can lead to significant improvements in T1DM-induced muscle atrophy and later improve the ability of skeletal muscle to utilize glucose to stabilize blood glucose concentration. These effect’s mechanisms may be associated with the MSTN/Akt/mTOR and Foxo1 pathway in the skeletal muscle.

Streptozotocin (STZ), a betacytotoxic agent, is a powerful alkylating agent that induces deoxyribonucleic acid breaks in pancreatic β cells, which subsequently induces type 1 (insulin-dependent) diabetes [33–35]. After 2 weeks of STZ administration, remarkable hyperglycemia and weight loss were observed, indicating that the pancreatic β cells were destroyed. It was reported that the withdrawal of insulin treatment in type 1 diabetic subjects causes a highly catabolic state characterized by an increased protein degradation rate that produces an accelerated loss of muscle mass, leading to muscle atrophy [30, 36, 37]. Moreover, in STZ-induced diabetes, autophagy is activated, which also contributes to the loss of muscle mass [38]. Insulin deficiency has been considered the main cause of muscle atrophy in subjects with type 1 diabetes, as insulin treatment prevents muscle loss [39]. In agreement with the previous studies, our results showed that STZ administration caused decreased muscle mass, strength, and fiber cross-sectional area, and insulin injection partially inhibited the STZ-induced weight loss and the decreases in muscle mass, strength, and fiber cross-sectional area. However, 6 weeks of LIPUS treatment also significantly improved the body weight, muscle mass, strength, and fiber cross-sectional area, similar to the insulin injection. Furthermore, the hyperglycemia was partially suppressed when exposed to LIPUS for 6 weeks. The mRNA and protein expression of GLUT-4, which reflects the ability of glucose to dispose in the skeletal muscle, were upregulated after 6 weeks of LIPUS treatment. These results indicate that LIPUS may contribute to improve T1DM-induced skeletal muscle atrophy and later elevate the glucose transport capacity of skeletal muscle. Furthermore, the LIPUS effects of reduced blood glucose concentration and inhibited muscle atrophy may be comparable to those of insulin.

MSTN is a key negative regulator of skeletal muscle development and causes muscle loss [40]. Activated MSTN binds to its receptor, ActRIIB, with high affinity and regulates the expression of target genes through the TGF-ß signaling pathway [41]. Furthermore, MSTN is able to inhibit Akt phosphorylation and thus downregulate the PI3K/AKT hypertrophy pathway [42]. According to our results for the MSTN/Akt signaling pathways, both the protein and mRNA expression of MSTN in muscles were decreased by LIPUS in diabetes rats, indicating that the inhibition of MSTN may contribute to improvements in T1DM-induced skeletal muscle atrophy. The recent research shows that MSTN is a secreted signaling molecule that not only acts to limit muscle mass but also circulates in the blood, where it acts as an endocrine factor [43]. Systemic overexpression of MSTN in adult mice was found to induce profound muscle and fat loss, analogous to that seen in human cachexia syndromes [44]. Moreover, local MSTN inhibition by overexpression of its propeptide increases glucose transporter expression and enhances skeletal muscle glucose disposal [45]. Although the serum MSTN concentration was not evaluated in this study, our previous study published in 2014 [46] showed that the changes in muscle MSTN could cause changes in serum MSTN. Therefore, in this study, local MSTN inhibition by local calf muscle stimulation could have a systemic effect, like on blood glucose and body weight, through its endocrine modes.

Earlier studies showed that mTOR activated by Akt leads to the activation of the pathways that promote protein synthesis and the inactivation of glycogen synthase [24, 47]. Akt also inactivates FoxO1 to inhibit muscle atrophy [25]. Hence, both the Akt/mTOR and Akt/FoxO1 pathways are important for muscle hypertrophy and atrophy. To examine more distal steps in the signaling pathway through MSTN/Akt, the effects of LIPUS on the expression of Akt-activated downstream targets, including mTOR and FoxO1, were analyzed. The results showed that 6 weeks of LIPUS treatment markedly activated AKT, upregulated mTOR expression, and downregulated FoxO1 expression. This suggests that MSTN may play an important role in LIPUS improving the T1DM-induced muscle atrophy by the Akt-independent increase in mTOR and inhibition of FoxO1.

## Conclusions

This study demonstrated that local LIPUS stimulation not only inhibits muscle atrophy but can also effectively reduce blood glucose concentration in type 1 diabetic rats. The MSTN/Akt/mTOR&FoxO1 signaling pathway may participate in this progress. Nevertheless, further research is warranted to elucidate the exact role of this pathway in this process. This study reveals that LIPUS may be used as an adjuvant therapy for T1DM patients with insulin or as a replacement therapy for those patients who are not suitable for insulin administration.

## Abbreviations

ActRIIB: Activin receptor type IIB
Akt: Protein kinase B
DC: Diabetic control group
DI: Diabetic insulin-treated group
DL: Diabetic LIPUS therapy group
FoxO1: Forkhead box protein O1
LIPUS: Low-intensity pulsed ultrasound
MSTN: Myostatin
mTOR: Mammalian target of rapamycin
NC: Normal control group
STZ: Streptozotocin
T1DM: Type 1 diabetes mellitus
TGF-β: Transforming growth factor-β
